# Comparative biochemical analysis of platelet-rich plasma-chitosan and platelet-rich fibrin-chitosan for treating tibial bone defects in rabbits

**DOI:** 10.14202/vetworld.2024.2036-2043

**Published:** 2024-09-13

**Authors:** Sajjad Javaid, Hamad Bin Rashid, Ayesha Safdar, Mamoona Chaudhry

**Affiliations:** 1 Department of Veterinary Surgery, University of Veterinary and Animal Sciences, Lahore, Punjab, Pakistan; 2 Department of Epidemiology and Public Health, University of Veterinary and Animal Sciences, Lahore, Punjab, Pakistan

**Keywords:** chitosan, fractures, platelet-rich plasma, platelet-rich fibrin, rabbits, tibia

## Abstract

**Background and Aim:**

Fracture healing can cause serious complications, both preoperatively and postoperatively, including malunion or non-union. Biomaterials can enhance the fracture healing process. This study aimed to compare platelet-rich plasma (PRP)-chitosan and platelet-rich fibrin (PRF)-chitosan on the basis of biochemical parameters for fracture treatment in rabbits.

**Materials and Methods:**

This study involved 12 clinically healthy rabbits. After preparing PRP and PRF, a 3-mm bone defect was created in the tibia of each rabbit. The animals were divided randomly into two groups (A and B). Group A received PRP-Chitosan, and Group B received PRF-Chitosan. Bone healing was assessed using biochemical parameters (calcium [Ca], phosphorus [P], serum alkaline phosphatase [ALP], and osteocalcin [Ocn]) at 2-, 4-, 6-, and 8-week postoperatively. The data were compared using repeated-measures analysis of variance (p < 0.05) with Statistical Package for the Social Sciences statistical software.

**Results:**

Group-wise comparison showed no significant differences (p > 0.05) between the groups, except for ALP levels, which were significantly higher in Group B than in Group A (p < 0.05). In the week-wise comparison, there was a significant difference between both groups, as Ca and ALP levels showed significant differences at all weeks postoperatively, whereas Ocn showed a significant difference at 2- and 4-week postoperatively (p < 0.05). However, there was no difference in P levels between the groups at any post-operative week (p > 0.05).

**Conclusion:**

Both combinations enhanced bone regeneration. However, PRF-Chitosan is a better combination for bone repair than PRP-Chitosan. There were some limitations of this study, such as a small sample size, only male rabbits were used, and a lack of mechanical testing; these limitations should be addressed in future studies. The insights gained from the present study may open a new approach to the use of a combination of biomaterials for bone healing, which should be further investigated clinically and in other animal models as a future scope.

## Introduction

Rabbits are small animals and have been used in many studies, including cardiovascular, physiological, and orthopedic studies. The skeleton of a rabbit is fragile; it accounts for almost 8% of its total body weight and is prone to injury [1]. One of the leading causes of disability is skeletal injuries, and one of the major types of skeletal injuries is bone fracture. This condition causes short-term morbidity but leads to long-term localized complications and can ultimately lead to delayed union or non-union. The timing and method of fracture fixation and its management remain controversial [2]. Different types of grafts are used to accelerate bone healing. The most widely used graft is an autologous bone graft or autograft that is removed from the ilium and is considered the gold standard due to its osteogenic, osteoinductive, and osteoconductive properties. However, the use of an autograft taken from the ilium or any other donor site is not always possible because of the limitations of bone stock, the need for a second surgical procedure, and possible complications, such as pain, infection, additional scarring, and the risk of local sensitivity alteration. Therefore, there is a need to develop more efficient, safer, and effective surgical procedures for bone repair. Thus, new technologies and advancements are continually being added to the therapeutic options of orthopedic surgeons [3].

Platelet-rich plasma (PRP) is a platelet-rich concentrate of plasma taken from the blood in a small volume. It comprises 95% platelets, 4% red blood cells (RBCs), and 1% white blood cells [4]. PRP is a relatively easy and inexpensive method for obtaining many important growth factors involved in angiogenesis, macrophage activation, mitogenesis, tissue repair, and regeneration, and reducing post-operative complications [5]. Moreover, these growth factors play important roles in the proliferation, differentiation, chemotaxis, and activity of bone cells during remodeling and fracture healing. In addition to these growth factors, there are many bioactive proteins which are responsible for attracting mesenchymal stem cells, osteoblasts, and macrophages, which help in removing the dead degenerated tissue, enhance tissue healing and regeneration. Platelets present in PRP accelerate epidermal, epithelial, and endothelial regeneration. They also increase collagen synthesis, angiogenesis, and hemostatic response to injury and reduce dermal scarring [4]. Besides these protein there are also some other proteins, such as fibrin, vitronectin, and fibronectin; these proteins increase cell adhesions, which act as a matrix for connective tissue, epithelium, bone formation, and wound healing. The use of PRP in veterinary medicine is of great interest for tissue repair [5].

Platelet-rich fibrin (PRF) is a second-generation platelet derivative. It is prepared by slow centrifugation without the addition of additives. It consists of a fibrin matrix, which is a derivative of a plasma molecule, fibrinogen. The molecules present in plasma help to coagulate during hemostasis. A fibrin monomer is formed when thrombin acts on fibrinogen, which polymerizes with other fibrin molecules to form fibrin fibers and helps in healing. PRF is an autologous product; thus, it minimizes the risk of immunological reactions and cross infections. The alpha (α) granules of platelets in PRF contain numerous growth factors that play important roles in the initial healing phase [6]. PRF comprises a tetra molecular structure containing stem cells, platelets, and cytokines. It acts as a biodegradable scaffold, facilitates microvascularization, and guides the migration of epithelial cells to its surface. PRF has great healing potential in soft tissues and bone without causing any inflammatory reactions and can be used alone or in combination with other bone grafts. PRF promotes bone growth, maturation, and hemostasis. PRF was first used in oral surgery [7] and is considered a new generation of platelet concentrate. It has been demonstrated in *in vitro* studies that PRF has great potential for the stimulation, proliferation, and differentiation of osteoblasts [8].

Chitosan is a naturally occurring biopolymer derived from chitin. It is biocompatible, and because of its porous microarchitecture, it can be used to prepare structures and scaffolds. It can also combined with other biomaterials to enhance bone healing. Chitosan is biodegradable, and adhesive in nature and has been widely used in many fields, such as tissue engineering, pharmaceutical applications, biomedicine, and surgery [9]. Nowadays, the pharmacological stimulation of fracture healing is gaining more attention. Biomaterials such as chitosan are used more frequently because they are non-living materials that can be used in biomedical, medical, and many other fields. In addition to that, the risk of immune-mediated rejection or disease transmission is minimized. Chitosan stimulates the proliferation and maturation of osteoblasts, which play critical roles in bone formation. It also minimizes the inflammatory response after implantation. These findings suggest that chitosan is a potential candidate for treating bone diseases such as bone defects and fractures [10].

The bone healing process is evaluated based on different biochemical parameters that fluctuate during the healing process, such as calcium (Ca), which is one of the main minerals present in bone, supports the skeletal system, and plays an important role in many physiological processes, including bone repair [11]. The second most abundant mineral after Ca is phosphorus (P), and its level changes during the bone healing [12]. In addition to these minerals, there are some proteins and enzymes whose levels also vary in bone healing, such as osteocalcin (Ocn) and alkaline phosphatase (ALP). Ocn is produced by osteoblasts [13], whereas ALP is produced by the liver and osteoblasts [11]. Therefore, by measuring the levels of the above-mentioned biomarkers, the bone healing process can be evaluated.

Developing new therapeutic modalities for fracture treatment is essential to improve bone healing outcomes. The treatment regimes currently in use often cause long-term morbidity and postoperative complications that can ultimately delay the process of bone repair. Therefore, by adding advanced therapies to the arsenal of veterinary orthopedics, we can reduce the risk of unwanted side effects, shorten recovery times, and improve bone regeneration. Both PRP and PRF are rich sources of growth factors that accelerate bone reconstruction and healing. Chitosan is biodegradable, non-toxic, hydrophilic, and biocompatible in nature and can be combined with PRP and PRF to increase their effectiveness and bioavailability of growth factors for longer periods. These treatments can ultimately reduce the duration of bone healing and complications during fracture healing.

Therefore, the present study aimed to compare the efficacy of PRP-chitosan and PRF-chitosan based on biochemical parameters: Ca, P, Ocn, and serum ALP for the treatment of bone defects in rabbits.

## Materials and Methods

### Ethical approval

This study was conducted under the guidelines issued by the ethical committee of University of Veterinary and Animal Sciences (UVAS) No. (DR/106) Dated 20/02/2023.

### Study period and location

The study was conducted from August 2023 to October 2023 at the Department of Veterinary Surgery (DVS), UVAS.

### Experimental animals

In this study, 12 healthy, active, adult male rabbits weighing 1.5–2.5 kg, regardless of breed, were bought from a local market in Lahore at least 14 days before surgery. Preoperatively, all basic health parameters; including temperature, pulse, respiration, and biochemical profile (Ca, P, Ocn, and ALP level) were recorded. In addition to that, X-rays were also performed to rule out any bone abnormalities.

Rabbits were randomly divided into two treatment groups; (A and B) with six rabbits per group. Group A received PRP + chitosan, and Group B received PRF + Chitosan.

### Bone defect model

**Figure-1:**
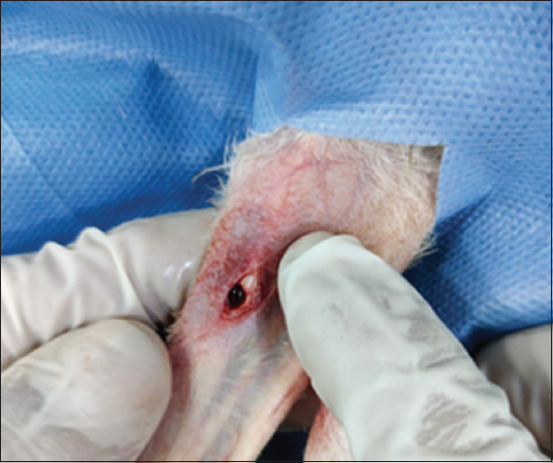
Three mm drill hole in rabbit tibia.

Rabbits were anesthetized using a cocktail of xylazine (Xylaz, FARVET Laboratories, Handelsweg, Netherlands) and ketamine (Ketalite, Elite Pharma Pvt. Ltd., Pakistan) through the intramuscular route at dose rates of 5 and 35 mg/kg, respectively. The medial surface of the right tibia of each rabbit was prepared aseptically for surgery, and an incision was made on the skin of the medial surface of the tibia. After controlling localized bleeding, the tibia was exposed, and a 3-mm hole was drilled in the midshaft of the tibia (Figure-1). The desired combination (PRP + Chitosan, PRF + Chitosan) was then applied to the bone defect site. The skin was closed in a standard fashion with the help of a simple interrupted suture pattern. Postoperatively, each rabbit was administered tramadol (Tramal, Searle Pakistan Pvt. Ltd., Pakistan) at a dose rate of 10 mg/kg for pain relief.

### PRP preparation

The rabbits were positioned sternally to visualize the jugular vein with the maximum dorsal extension of the neck and 8 mL blood was collected using a 10 mL syringe. The sample was then transferred into two sterile tubes containing sodium citrate (anticoagulant), and a third tube coated with ethylenediaminetetraacetic acid (anticoagulant) was filled with 0.8 mL of the sample for platelet counting. After confirming that the platelets were within the normal physiological range, preparation for PRP was started.

For PRP preparation, tubes were centrifuged twice; the first centrifugation was performed at 372× *g* for 10 min, resulting in three layers: plasma at the top, buffy coat in the middle, and RBCs at the bottom. The top layer containing plasma was transferred into another sterile tube, and a second centrifugation was performed at 581× *g* for 10 min, resulting in two layers. The top layer was platelet-poor plasma (PPP), and the bottom layer was the PRP button. The PPP was removed from the top to ensure that only about 1 mL was left in the tube, and the bottom was agitated to suspend the platelets, resulting in PRP. Subsequently, platelets were counted in the samples to determine whether the platelet count was greater after processing. Then, it was activated by adding 0.3 mL of Ca gluconate to form a PRP gel. In the present study, the average platelet concentration in whole blood was 386,000/μL (normal physiological range 290,000–678,000) and the average platelet concentration in PRP was 18,21,206/μL which is in accordance with the range (470,000/μL–44,20,000/μL) [14].

### PRF preparation

From the jugular vein of the rabbit, 8 mL of blood was collected in sterile tubes. Blood was then centrifuged at 1308× *g* for 10 min, resulting in three distinct layers. The uppermost layer was PPP or a-cellular plasma. The central and bottom layers were the fibrin and RBCs, respectively. The PPP was discarded, and the central layer was carefully removed using toothed tissue forceps. To remove the exudate trapped in the PRF clot, it was placed in a sterile container for 10 min to release the serum trapped in it [7]. Chitosan powder was purchased from a local market.

### Statistical analysis

The data collected from both groups are presented as mean ± standard deviation. The data were compared using repeated measure of one-way analysis of variance, p < 0.05 on SPSS statistical software 20 (IBM Corp., NY, USA).

## Results

### Ocn

**Figure-2:**
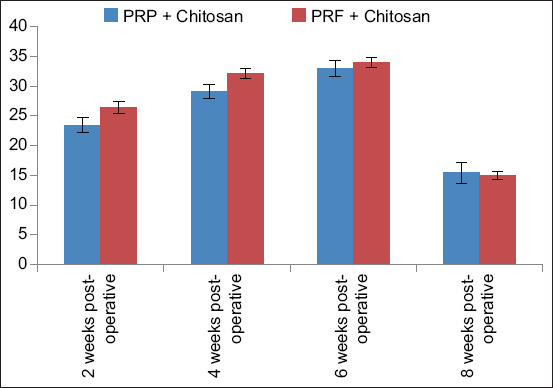
Osteocalcin levels in Groups A and B at 2, 4, 6, and 8 weeks after surgery.

Ocn levels were recorded in both groups at 2-, 4-, 6-, and 8-week postoperatively. In both groups, the Ocn level started to increase 2-week postoperatively and continued to increase until it reached its peak at 6-week postoperatively. In the overall comparison, there was no significant difference between the groups (Table-1 and Figure-2). However, in the *post hoc* test in a week-wise comparison, there was a significant difference in both groups at 2- and 4-week postoperatively as p < 0.05.

**Table-1:** Results of level of osteocalcin mean ± SD of both groups (A and B) at 2-, 4-, 6-, and 8-week postoperatively.

Groups	2-week post-operative	4-week post-operative	6-week post-operative	8-week post-operative
PRP + Chitosan	23.43 ± 1.27	29.11 ± 1.23	32.96 ± 1.34	15.40 ± 1.80
PRF + Chitosan	26.45 ± 0.98	32.15 ± 0.88	33.95 ± 0.82	14.95 ± 0.65

SD=Standard deviation, PRP=Platelet-rich plasma, PRF=Platelet-rich fibrin

### ALP levels

**Figure-3:**
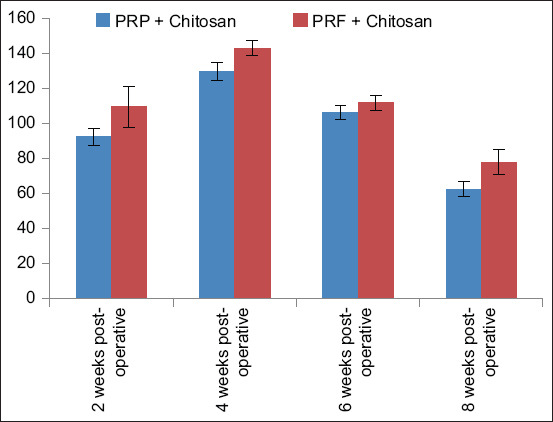
Alkaline phosphatase levels in Groups A and B at 2, 4, 6, and 8 weeks after surgery.

ALP levels were recorded in both groups at 2-, 4-, 6-, and 8-week postoperatively. The normal physiological range of ALP in rabbits is 12–96 IU/L [15]. After 2 weeks, serum ALP levels were within the normal range in Group A but increased in Group B, indicating increased ALP activity as bone repair was started. At 4-week postoperatively, peak levels were observed in both groups. At 6 weeks, the ALP level was still high in both groups, and at 8-week postoperatively, it returned to within the normal physiological range in both groups (Table-2 and Figure-3). In the overall comparison, there was a significant difference between both groups as p < 0.05. In a week-wise comparison, there was also a significant difference in both groups at all weeks postoperatively as p < 0.05.

**Table-2:** Results of the alkaline phosphatase mean ± SD of both groups (A and B) at 2-, 4-, 6-, and 8-week postoperatively.

Groups	2-week post-operative	4-week post-operative	6-week post-operative	8-week post-operative
PRP + Chitosan	92.38 ± 4.70	129.75 ± 5.14	106.47 ± 4.21	62.59 ± 4.30
PRF + Chitosan	109.59 ± 11.91	143.30 ± 4.36	111.96 ± 4.30	77.89 ± 7.17

SD=Standard deviation, PRP=Platelet-rich plasma, PRF=Platelet-rich fibrin

### Ca level

**Figure-4:**
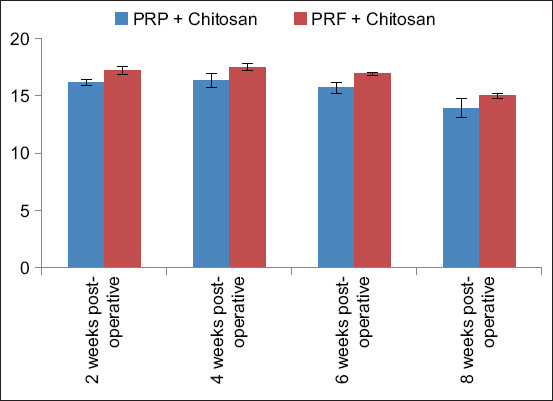
Calcium levels in Groups A and B at 2, 4, 6, and 8 weeks after surgery.

Ca levels were measured in both groups at 2-, 4-, 6-, and 8-weeks after surgery. After 2 and 4 weeks, the Ca level was higher in both groups than the normal physiological range of serum Ca levels in rabbits, which is 12.5–16 mg/dL [16]. Peak levels were observed in both groups at 4-week postoperatively. At 6-week postoperatively, Ca levels in Group A returned to the normal range, whereas in Group B, Ca levels remained high up to 6-week postoperatively and then returned to normal at 8-week postoperatively (Table-3 and Figure-4). In the overall comparison, there was no significant difference between both groups (p > 0.05). However, in the week-wise comparison, there was a significant difference between the groups at all weeks (p < 0.05), and the highest significant difference was observed at 2- and 6-week postoperatively.

**Table-3:** Results of level of calcium mean ± SD of both groups (A and B) at 2-, 4-, 6-, and 8-week postoperatively.

Groups	2-week post-operative	4-week post-operative	6-week post-operative	8-week post-operative
PRP + Chitosan	16.16 ± 0.26	16.31 ± 0.62	15.68 ± 0.48	13.90 ± 0.81
PRF + Chitosan	17.21 ± 0.34	17.48 ± 0.30	16.90 ± 0.10	15.00 ± 0.23

SD=Standard deviation, PRP=Platelet-rich plasma, PRF=Platelet-rich fibrin

### P level

**Figure-5:**
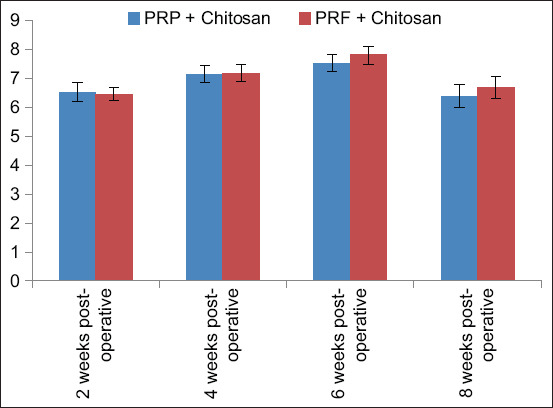
Phosphorus levels in Groups A and B at 2, 4, 6, and 8 weeks after surgery.

P levels were recorded in both groups at 2-, 4-, 6-, and 8-week postoperatively. The normal physiological range of P in rabbits is 4–6.5 mg/dL [15]. At 2-week postoperatively, Group A had more P levels than Group B, and both groups showed high P levels at 4- and 6-week postoperatively, with peak levels observed at 6 weeks postoperatively (Table-4 and Figure-5). In the overall comparison, there was no significant difference between the groups (p > 0.05). In the week-wise comparison, there was no significant difference (p > 0.05).

**Table-4:** Results of level of phosphorous mean ± SD of both groups (A and B) at 2-, 4-, 6-, and 8-week postoperatively.

Groups	2-week post-operative	4-week post-operative	6-week post-operative	8-week post-operative
PRP + Chitosan	6.53 ± 0.32	7.15 ± 0.31	7.53 ± 0.29	6.38 ± 0.39
PRF + Chitosan	6.46 ± 0.22	7.18 ± 0.29	7.81 ± 0.31	6.68 ± 0.37

SD=Standard deviation, PRP=Platelet-rich plasma, PRF=Platelet-rich fibrin

## Discussion

The skeleton system is unique, unlike most other systems, as it is composed of mainly calcified tissue; the bone [17]. The treatment of bone defects is always challenging in veterinary orthopedics because of the many post-operative complications. Different biomaterials have been used in different models for the evaluation of bone repair, as reported by many researchers, to explore their bone-healing potential [18]. In the present study, chitosan was used along with autologous PRP and PRF in experimentally induced tibial bone defects in rabbits.

Chitosan is derived from chitin through deacetylation. It is generally used as a biomaterial, especially in drug-delivery systems, and can be used in combination with other materials to enhance their therapeutic efficiency [19]. In the present study groups, the animals treated with a combination of chitosan showed no clinical signs of inflammation or infection. This may be attributed to the unique biocompatibility, facile modification, versatility, biodegradability, antibacterial, hydrophilic, and non-toxic properties of chitosan [20].

The most applicable biomaterial among the different biomaterials that are used to evaluate bone healing is PRP. Because it is prepared using a minimally invasive technique, rich in growth factors, and increases the healing process [3]. It is now well established that PRP increases bone healing both alone and in combination, as reported by many researchers, such as Batista *et al*. [3], who reported that the group treated with PRP had greater cortical bone thickness and consolidation in rabbits. In previous studies, PRP was used along with bone graft materials. As reported by Kanthan *et al*. [21], PRP increased bone healing when used in combination with bone grafts in comparison to PRP alone. However, its potential to increase bone healing in combination with other biomaterials, such as chitosan, remains under consideration. As reported by Bakhtiarimoghadam *et al*. [22], PRP along with chitosan can be a novel approach for bone tissue engineering.

The other biomaterial used in this study was PRF. It is a second-generation platelet derivative discovered by Choukroun *et al*. [23] with a much simpler preparation method than PRP gels, as no activator is required. PRF, in combination with biomaterials, accelerates bone healing, as in this study, PRF was used in combination with chitosan. At present, the potential of bone regeneration with this combination is still underexplored, as very few researchers have conducted research using PRF and chitosan in combination. As reported by Rastegar *et al*. [24], PRF-loaded chitosan scaffolds can enhance bone regeneration with improved osteogenic potential. Similarly, it was reported by Chen *et al*. [25] that dual-function injectable fibrin gel incorporated with sulfated chitosan nanoparticles can be used for the treatment of fractures in postmenopausal mice with osteoporosis because it enhances bone healing.

Ocn is one of the most abundant non-collagenous proteins in bone, expressed in osteoblasts. An increase in the activity of Ocn mRNA can promote the bone formation process. Ocn is one of the hormones that regulate the activity of osteoblasts, osteoclasts, and adipocytes, and in addition to that, it also controls bone production [26]. In the present study, PRP was used in combination with chitosan, and it improved bone healing. However, many researchers used this combination only *in vitro*, as it was reported by Shimojo *et al*. [27] that the composite of PRP and chitosan improved the performance of PRP and its regeneration ability. Similar findings were reported by Huang *et al*. in [28], who reported that the combination of PRP and chitosan scaffold has a synergistic effect on bone healing in rabbit radial defects, as evaluated by gross, radiological, and histological examination. However, immunohistochemicals showed no difference between individual and composite groups. In this study, only combinations were studied, not the individual group. However, some findings reported by Huang *et al*. [28] are different from the present study, as the examination was carried out after 12 weeks only, whereas in the present study, it was carried out every 2 weeks up to 8-week postoperatively and it showed an increase in bone regeneration. Similarly, Ansarizadeh *et al*. [29] reported that the addition of PRF in collagen-chitosan showed better osteogenic differentiation. Both combinations (PRP + chitosan, PRF + Chitosan) showed better Ocn activity. This might be because PRP and PRF contained high concentrations of platelets and growth factors. Moreover, the combination of PRP and PRF with chitosan sustainedly released growth factors for longer periods. The addition of chitosan to PRP increases the life span and secretion of growth factors as long as up to 24 days. Moreover, this combination also increases the number of osteoblasts [30].

ALP is a common molecular marker of osteogenic differentiation. ALP is primarily secreted by bone and liver. Therefore, elevated ALP levels are an indication for bone or liver disorders. In bone, ALP is secreted in large amounts by osteoblasts, which play important role in matrix production and bone mineralization by locally increasing Ca phosphate concentrations. It is believed that ALP boosts the levels of inorganic pyrophosphate or inorganic phosphates in the affected area, both of which are essential for bone healing [31]. PRP + chitosan can promote bone healing as Group A showed increased ALP during fracture healing. Similar findings were reported by Shimojo *et al*. [27], who showed that the composite of PRP and chitosan showed better osteogenic differentiation than PRP alone. Similarly, it was reported by Bi *et al*. [32] that the ALP level was higher in the group treated with the combination of PRP and chitosan than in the group treated with chitosan alone. In the current study, Group B showed a higher ALP value than Group A, with peak values at 4 weeks and remained high up to 6-week postoperatively. Similar findings were reported by Sidharta *et al*. [33], who reported that the combination of PRF with chitosan resulted in better ALP levels than the PRF and the control groups. The increased ALP levels were primarily due to increased chondroblast proliferation during bone fracture repair. Moreover, periosteum breakdown also increases serum ALP levels [31].

Ca is one of the major minerals of bone. It is present in the form of Ca hydroxyapatite crystals in bone and plays a key role in bone metabolism and mineralization. It is well-established that Ca is one of the most important nutrients for bone healing [34]. One of the major indicators of fracture healing in animals is the evaluation of serum Ca levels. Ca deficiency suppresses bone formation and increases Ca mobilization from bones [14]. In Group A, serum Ca levels increased at 4 weeks postoperatively. This finding could be due to the presence of large amount of platelets and proteins present in PRP. Moreover, because of sticky clot formation due to the presence of platelets, it also increases the angiogenesis and mitogenesis at the healing site. In Group B, serum Ca levels were high 4-week postoperatively. Similar findings were reported by Sheu *et al*. [34], who reported that Ca levels were high in all treatment groups, including the PRF group and their combinations, except for the control group. The initial increase and subsequent decrease in serum Ca levels are primarily attributed to crystalline salt deposition in the organic matrix, which is mainly composed of Ca and phosphatase [31].

P is involved in different biological processes, such as the conduction of nerve stimuli, nucleic acids, bone-building materials, and regulatory roles in osteolysis as well as in osteogenesis. It is second next basic component of bone tissue after Ca [35]. In this study, PRF, along with chitosan, increased P levels. Similar findings were reported by Sheu *et al*. [34], who found that 8 weeks after transplantation, the P level was high in the group treated with the combination of PRF. These findings coincide with the present study as serum P levels continued to increase in Group B in which PRF was used along with chitosan for up to 6-week postoperatively. It was also reported by Gharib *et al*. [36] that chitosan scaffolds have a positive effect on Ca and P levels during bone healing. In addition, it provides cell adhesion, migration, proliferation, growth, and porosity for rapid bone repair.

Similarly, it was reported by Nazht *et al*. [37] that Ca and P levels were increased during fracture healing in rabbits. Group A showed an increase in serum P level, but the increase was not as high as in Group B. The combination of PRP and chitosan has a synergistic effect on healing. This could be due to the fact that chitosan can provide an environment that increases osteogenic and angiogenic cell function. Moreover, chitosan increases the contact between platelet lysate and injured tissue, thereby extending its therapeutic effect [38].

It was reported by Segundo *et al*. [39] that the combination of PRP and chitosan promoted better bone and cartilage healing. Similarly, it was reported by Pritia *et al*. [40] that the combination of PRF and chitosan increased the migration and proliferation of osteoblast cells, but this was an *in vitro* study. Kim *et al*. [41] also reported that the group treated with PRF had increased bone volume and increased bone mineral density after 6 weeks of calvaria defect in rabbits, and the effects of PRP and PRF on bone healing were similar. Therefore, the combination of these biomaterials can be used to increase bone healing in rabbits.

## Conclusion

The preparation of PRP and PRF is a simple and inexpensive technique that can benefit veterinary surgeons in increasing the bone healing process. Based on the current study, we recommend using the PRF/chitosan combination to treat bone defects. This combination introduces a new modality for treating bone defects that can reduce bone healing time and complications during bone repair. In the future, a combination of biomaterials (PRP + Chitosan, PRF + Chitosan) should be used in clinical settings and other animal models for further evaluation.

## Authors’ Contributions

SJ: Conceptualized and designed the study, drafted the manuscript, investigation, and methodology. HBR: Conceptualized the study, drafted, reviewed, and edited the manuscript, and validation. AS: Methodology, reviewed and edited the manuscript, and data curation. MC: Reviewed the manuscript, software, and formal and statistical analysis. All authors have read and approved the final manuscript.
